# Difficult colonoscopies in the propofol era

**DOI:** 10.1186/1471-2482-12-S1-S9

**Published:** 2012-11-15

**Authors:** Fabrizio Cardin, Nadia Minicuci, Federico Campigotto, Alessandra Andreotti, Elisa Granziaera, Barbara Donà, Bruno Martella, Claudio Terranova, Carmelo Militello

**Affiliations:** 1 Department of Surgical and Gastroenterological Sciences, Padova University Hospital, Italy (Via Giustiniani n.2, 35126 Padova, Italy; 2 National Council Reaserch, Institute of Neuroscience, Padova, Italy (Via Cesare Battisti n.206, 35124 Padova, Italy; 3 Istituto Oncologico Veneto, IRCCS, Padova, Italy; 4 Legal Medicine Unit, Department of Molecular Medicine, Padova University Hospital, Italy (Via Gabelli n.63, 35121 Padova, Italy

## Abstract

**Background:**

To study the relationship between endoscopic practice and adverse events during colonoscopy under standard deep sedation induced and monitored by an anesthetist.

**Methods:**

We investigated the routine activity of an endoscopy center at the Padova University teaching hospital. We considered not only endoscopic and cardiorespiratory complications, but also the need to use high-dose propofol to complete the procedure, and the inability to complete the procedure. Variables relating to the patient’s clinical conditions, bowel preparation, the endoscopist’s and the anesthetist’s experience, and the duration of the procedure were input in the model.

**Results:**

617 procedures under deep sedation were performed with a 5% rate of adverse events. The average dose of propofol used was 2.6±1.2 mg/kg. In all, 14 endoscopists and 42 anesthetists were involved in the procedures. The logistic regression analysis identified female gender (OR=2.3), having the colonoscopy performed by a less experienced endoscopist (OR=1.9), inadequate bowel preparation (OR=3.2) and a procedure lasting longer than 17.5 minutes (OR=1.6) as the main risk factors for complications. An ASA score of 2 carried a 50% risk reduction (OR=0.5).

**Discussion and conclusions:**

Our model showed that none of the variables relating to anesthesiological issues influenced which procedures would prove difficult.

## Background

A thorough colonoscopy is the endoscopist’s most important goal, especially in screening programs. Failure to complete a caecal intubation may be related to technical issues, the patient’s tolerance, and/or bowel preparation. Various scientific societies have recommended programs designed to improve the endoscopists’ performance [1,2] and several bowel preparation methods have been investigated to facilitate patient compliance with the colon cleansing requirements [3].

Among all the factors capable of influencing the colonoscopy success rate, increasing the patient’s tolerance by means of a pharmacological sedation is the one that enables the best results to be achieved. On the other hand, such an approach has raised major concern regarding: a) the generic risk relating to the use of sedatives, e.g., respiratory depression and cardiovascular complications, compounding the specific risk of perforation or bleeding with possible related medico-legal consequences[4]; b) the involvement of different health professionals in the endoscopy room; c) the additional costs for monitoring devices and ancillary personnel [5]. All these reasons make the use of deep sedation debatable and many issues remain to be solved or need further investigation [6,7].

Guidelines have been developed on the factors to consider in order to guarantee a safe procedure, since adverse events may depend not only on the endoscopist, but also on the activities of the anesthetist, including the preliminary risk assessment and patient monitoring [8,9]. The use of deep sedation with propofol in colonoscopy is therefore a controversial issue because it demands careful planning to maximize patients’ tolerance and minimize their risks for diagnostic, or relatively simple therapeutic procedures (such as polypectomy).

Since the routine use of deep sedation with propofol is relatively rare, only a few studies have addressed the correlations between the factors that might contribute to ensuring a risk-free, effective and tolerable colonoscopy.

The aim of our study was to investigate whether the critical situations encountered in the colonoscopy suite or incomplete deep sedation-colonoscopies can be predicted from the endoscopic or anesthesiological practices involved and/or from the patient’s condition.

We considered a procedure as “difficult” if the colonoscopy was incomplete or complicated for endoscopic reasons, or for reasons relating to the anesthetist, or to hemodynamic/respiratory changes in the patient, or if the procedure required a dose of propofol higher than was routinely used, with the risk of inducing general sedation.

## Methods

We investigated the routine activity of an endoscopy center at the Padova University teaching hospital, consecutively enrolling in the study all patients who underwent a colonoscopy under propofol sedation over a period of six months.

Subject to patients’ informed written consent, their clinical history was recorded to identify any anesthesiological or procedural risks, collecting data on: a) non-gastrointestinal conditions; b) allergies or side effects of previously-used anesthetics; c) ASA status. Information was subsequently recorded on the completeness of the colonoscopy, the propofol dosage used and any adverse events on a case report form (CRF) formulated specifically for the purposes of the study. The health professionals responsible for compiling the CRF were unaware that they were participating in the study.

### Classification of health professionals

The gastroenterologists and anesthetists involved in the endoscopies were classified according to their level of experience.

Gastroenterologists were grouped into three categories: 1) the “less experienced non-specialists” if they had been performing endoscopies for less than 10 years; 2) the “more experienced non-specialists” if they had been performing endoscopies for more than 10 years but were not dedicated exclusively to endoscopic activities; and 3) the “highly-experienced specialists” if they had been performing endoscopies for more than 10 years and handled at least four endoscopic sessions a week.

Anesthetists were classified as “juniors” (if they had practiced as anesthesiologists for less than 2 years), “seniors” (2-5 years in practice) and “experts” (more than 5 years in practice).

### Propofol sedation

Before the colonoscopy, all patients received an initial induction dose of propofol to induce a lethargic response to oral stimuli and the absence of any corneal reflex. During the procedure, propofol was titrated by the anesthetist with intermittent boluses if patients showed signs of more than mild discomfort and occasional grimacing, becoming agitated or clearly in pain at every stage of the procedure.

Patient monitoring was started before sedation and continued until patients recovered to check for any episodes of hypotension, hypoxemia, cardiac arrhythmia, which were recorded in the CRF and treated pharmacologically, where necessary.

The patients’ preparatory colon cleansing consisted in their ingesting 4 liters of Macrogol solution at home the day before the colonoscopy. Their bowel preparation was classified as “adequate”, “with residual matter” or “inadequate”.

### Definition of a difficult procedure

A procedure was defined as being “difficult” when:

1. a total dose of propofol/kg 1 standard deviation above the mean was administered;

2. the procedure met with complications, such as perforation or bleeding;

3. the colonoscopy was incomplete for technical reasons (other than a patient’s inadequate bowel preparation);

4. there were adverse events relating to the sedation, e.g., episodes of apnea, hypo- or hypertension, hypoxemia, or cardiac arrhythmia.

### Efficacy endpoints

The main efficacy endpoint was the completeness of the colonoscopy, i.e. the identification of a normal cecum anatomy or ileocolic anastomosis, without the need for any anesthesiological, pharmacological or manual intervention to deal with respiratory or cardiocirculatory complications. Colonoscopies stopped due to the diagnosis of a pathological stenosis considered as completed procedures.

Hemodynamic monitoring data were used to determine the duration of the colonoscopy.

### Statistical analysis

Summary statistics - mean values or percentages - were compared by age group using the t-test or chi-square test, respectively. Otherwise, analogous non-parametric tests were used (the Wilcoxon rank-sum test or Fisher’s exact test). Predictors of a “difficult procedure” were investigated by multivariate stepwise (p-entry=0.15) logistic regression analysis including all variables mentioned in table 3. The SAS statistical software, rel. 9.2 was used for the analysis.

## Results

During the study period, 617 colonoscopies were performed under deep sedation in patients, mean 61.1 years old (SD=13.2), 48.3% of them males.

Of the 14 endoscopists involved in these procedures, 5 were classified as with “less experienced” and performed 18.3% of the endoscopies, 6 were classified as “more experienced non-specialists” and performed 59.5% of the colonoscopies and the remaining 3 were classified as “highly-experienced specialists” and performed 22.2% of the procedures. Junior anesthetists (n=24) were involved in 67.6% of the procedures, seniors (n=13) in 26.7% and experts (n=5) in 5.7%.

The incomplete procedures (excluding those due to inadequate bowel preparation) amounted to 6.8% (42/617) (Table 1). There were 75 patients (12.2% of the sample) with ASA scores of 3 or 4. The average dose of propofol administered was 183±74.9 mg, while the average dose per kg of body weight was 2.6±1.2 mg/kg.

**Table 1:** Characteristics of the endoscopies.

Characteristics	Values (n=617)
Complete colonoscopy	93.2% (575/617)

Incomplete colonoscopy	6.8%(42/617)

technical reasons	76.2% (32/42)
inadequate bowel preparation	23.8% (10/42)

Complications	6.5% (40/617)

endoscopic	2/40
respiratory	8/40
hemodynamic	28/40
gastrointestinal	2/40

Procedures with polypectomy	15.7% (97/617)

ASA (%)	

1	36.6% (226/617)
2	51.2% (316/617)
3+4	12.2% (75/617)

Propofol (mg)	

mean±SD	183.3±74.9
range	20-580

Propofol (per kg of b.w.)	

mean±SD	2.6±1.2
range	0.2-9.8

Duration of procedure	

mean±SD	23.2±11.4
range	2-85

Tables 2 shows the characteristics of patients whose procedures were and were not difficult, and Table 3 shows the results of the logistic regression analysis.

**Table 2:** Distribution of the main characteristics by procedure (difficult vs routine).

	Difficult procedure (n=142)	Routine procedure (n=475)	P value
Gender (females)	66.2%	47.4%	<0.0001

Mean age±SD	58.8±14.6	61.8±12.6	0.03

Endoscopists			0.014

less experienced	26.8%	15.8%	
more experienced non-specialists	52.1%	61.7%	
highly experienced specialists	21.1%	22.5%	

Current diseases			

Allergy	20.4	22.1	0.72
Heart	16.9	15.2	0.60
Liver	7.7	5.3	0.30
Kidney	2.8	4.6	0.47
Thyroid	5.6	5.7	1.00
Polypectomy	14.8	16.0	0.79

Duration of colonoscopy (minutes)			

mean±SD	26.9±14.2	22.0±10.2	0.0002
range	5-85	2-65	

Bowel preparation			

adequate	62.7%	77.3%	<0.0001
with residual matter	26.1%	18.7%	
inadequate	11.2%	4.0%	

ASA score			0.003

1	46.5%	33.7%	
2	38.7%	54.9%	
3+4	14.8%	11.4%	

**Table 3:** Odds ratios and 95% CI for difficult procedures.

	Odds ratio	95% Confidence Interval	P value
Females	2.312	1.528-3.498	<0.0001

ASA=1	1		
ASA=2	0.539	0.344-0.844	0.0070
ASA=3,4	1.043	0.531-2.050	0.9023

Highly-experienced specialist	1		

Less experienced	1.925	1.046-3.541	0.0352

More experienced non-specialist	1.028	0.620-1.705	0.9151

Adequate bowel preparation	1		

Presence of residual matter	1.465	0.917-2.339	0.1099

Inadequate bowel preparation	3.208	1.511-6.808	0.0024

Duration of procedure ≥17.5	1.633	1.050-2.538	0.0294

In all, 142 procedures (23%) were classified as difficult. In addition to the procedures that were not completed (42) and those incurring complications (40) during the colonoscopy, 77 patients were given more than 3.8 mg/kg of propofol. Fifteen patients experienced more than one complication.At univariate level, we found a statistically higher prevalence of female gender (66.2%), “less experienced” endoscopists (26.8%), residual matter or inadequate bowel preparation (26.1% and 11.2%, respectively) and an ASA score of 1 (46.5%) in the group with difficult procedures, this group was also statistically younger (mean 58.8 years) and their procedure took longer (26.9 minutes).

The logistic regression analysis identified female gender (OR=2.3), having the colonoscopy performed by a less experienced endoscopist (OR=1.9), inadequate bowel preparation (OR=3.2) and a procedure lasting longer than 17.5 minutes (OR=1.6) as the main risk factors for complications. On the other hand, an ASA score of 2 carried a 50% risk reduction (OR=0.5). The logistic model had an area under the curve (ROC) of 70%. None of the other variables contributed to the predictive power of the model.

## Discussion and conclusion

It has been demonstrated that cardiorespiratory complications during a colonoscopy may be associated with anesthesiological practices (especially if sedation is kept constant using the simple propofol boli technique) due to an excessive dose of drug, inadequate patient monitoring, and/or an excessively rapid induction of sedation [10,11].

Our study shows that difficulties encountered during colonoscopy procedures cannot be explained, however, by variables relating to anesthesiological activity during deep sedation with propofol. There are also technical issues, already identified in the pre-propofol era [12], that contribute to complicating a colonoscopy.

Analyzing our black box warnings on deep sedation with propofol highlighted that the endoscopist’s experience, bowel preparation, and female gender are the factors that correlate with difficult procedures in patients sedated up to level 4 of the spectrum of continuum of sedation, as defined by the American Society of Anesthesiology. Interventions to restore adeguate airway function or ventilation are likely to be needed for this type of patient. Unsatisfactory bowel preparation, female gender, age and constipation were identified by Won Ho Kim [13] as determinants of difficulties encountered in performing colonoscopies, while Doger [14] found that gender, age and the endoscopist’s experience contributed to the duration of the procedure. These two studies report results similar to ours, though their patients were not administered propofol.

Some studies have tried to find strategies for containing the anesthesiological risk by reducing the propofol dosage, without increasing the patient’s discomfort, using either a combination of drugs [15] or a patient-controlled infusion [16]. Our study focused instead on adverse events and their causes, correlating these with the total dose of propofol administered by the anesthetist on the basis of evidence of the patient’s pain, a situation that can lead to over-sedation using the drug titers adopted by the anesthesist. That is why we included the use of a dose of propofol more than one standard deviation above the mean among the “difficult procedures”. Since a relationship has been shown between propofol dosage and cardiovascular events [10,17], high doses can be seen as indicators of situations at risk. Critical situations can crop up in the endoscopy room due to the need to take action to maintain the patient’s vital parameters. The average propofol dose used in our study was 2.8 mg/kg, which is higher than in previous studies [15,18] but analysis indicated that the anesthetist’s role had no influence on the rate of difficult procedures. One explanation for this may have to do with the endoscopist finding it difficult to complete quickly and as painlessly as possible due to inexperience or the need to perform a polypectomy.

The interaction between propofol dosage and the patient’s conditions was also studied, revealing negative effects [8]. In our multivariate analysis, an ASA score of 2 tended to protect against occurrence of difficulties; Heuss found [18] that the ASA factor has little influence on the secondary effects of sedation and there is only a limited risk of oxygen desaturation for ASA levels III and IV. The only patient-related variable affecting the rate of difficult procedures was gender, as in several other studies on “difficult” colonoscopies in the pre-propofol era [19,20].

One limit of our study is the lack of a control group undergoing colonoscopy without sedation, but we feel - given that all the variables that we considered were related to the endoscopic techniques, and in the light of comparisons drawn with other reports on colonoscopy performed without propofol - that even when patients are under deep sedation, the expertise of the endoscopist is still more important to the success of a colonoscopy than is the role of the anesthetist. Our study was designed on the strength of a discussion on endoscopic sedation policy issues, developed within the American Gastroenterology Association [21].

We did not consider aspects relating to the costs of the sedation procedure, since these are strongly influenced by local conditions within different public health systems and not easy to extend to an international scenario. It was recently pointed out that using propofol at endoscopy centers facilitates patient turnover [22]. Unfortunately, we are unable to provide information on our patients’ recovery and their compliance with the procedure because these data are not routinely collected at our center. We preferred to keep to the data collected in daily practice to avoid influencing the endoscopist’s routine and giving rise to any Hawthorne effect.

We also believe that the fact we used no pumps for drug infusion (leaving the propofol dosage up to the anesthetist) and that we assessed the activity of anesthetists and endoscopists with different levels of expertise constitutes an advantage in terms of the general applicability of our findings. These elements probably influenced endoscopic performance: our 93.2% success rate (94.8% if incomplete procedures due to inadequate bowel preparation are disregarded) is definitely higher than at other endoscopy centers where deep sedation is not used [23], getting closer to the target indicated for screening examinations [24].

Our findings could be compared (given their geographical and organizational affinity) with those reported by Fasoli [25], showing that propofol helps to improve the performance of less experienced endoscopists. We, indeed, confirm Karan’s comment [8] that a prolonged procedure represents further duties for the anesthesiologist; and according to Ju-Mei [16] the duration of the colonoscopy and the patient’s pain are reasons for the use of higher doses of propofol.

It has also been said that cardiopulmonary events account for about half of the adverse events during endoscopies [24]. In our series, we recorded one perforation, one hemorrhage caused by polypectomy and 36 cardiorespiratory event. This number is elevated because we considered not only events requiring tracheal intubation or cardiopulmonary resuscitation (which never proved necessary in our series), but also events requiring airway manipulation or pharmacological intervention by health professionals other than the endoscopist, involved in the procedure, since this obviously interfered with the endoscopic procedure proper. However, the 5% adverse events rate is comparable with the one recorded by Rex [17], who also showed that trained nurses and endoscopists can administer propofol safely for endoscopy. Our study, referred to an anesthesiologist-based standard endoscopic sedation, shows that adverse events always have to do with the endoscopic techniques, whereas the anesthetist’s experience had a negligible influence. Our findings seem to contradict any concern [26] that the use of anesthesiologists during colonoscopy has the potential to transform endoscopic practice.

## Competing interest

The authors declare that they have not competing interest.

## Authors’ contributions

FC, NM and CT designed the study. FC, EG and BA gave a fundamental contribution to the recruitment of patients and contributed to the literature review, to the writing and reviewing of the paper. NM and AA performed the statistical analysis and contributed to the writing of the manuscript. FCamp, EG and BD contributed to the interpretation of the data, to the writing and reviewing of the paper. CM and BM performed a critical revision of the article for important intellectual content.

All the authors read and approved the final manuscript.
